# Omental plug using double horizontal mattress suture for gastric perforation on elderly patients with boey score 3: A case series

**DOI:** 10.1016/j.amsu.2021.102647

**Published:** 2021-07-30

**Authors:** Adeodatus Yuda Handaya, Joshua Andrew, Ahmad Shafa Hanif, Kevin Radinal Tjendra, Azriel Farrel Kresna Aditya

**Affiliations:** Digestive Surgery Division, Department of Surgery, Faculty of Medicine, Universitas Gadjah Mada/Dr. Sardjito Hospital, Yogyakarta, 55281, Indonesia

**Keywords:** Boey score 3, Double horizontal mattress suture, Elderly, Gastric perforation, Omental plug

## Abstract

**Introduction:**

Gastric perforation is a life-threatening condition. Patients with gastric perforation with Boey score 3 has very high mortality rate. Immediate source control is required for primary repair and preventing further complications. Furthermore, elderly patients pose a greater risk of morbidity and mortality in cases of gastric perforation, especially during and after emergency surgery.

**Case presentation:**

We present two cases of elderly patients with gastric perforation with Boey score 3. We performed omental plugging technique with double horizontal mattress suture type. In these cases, we decided not to perform biopsy and margin freshening of the perforation.

**Discussion:**

We performed omental plugging technique because we are confident that it could cover the perforation completely without causing gastric outlet obstruction. An emergency source control surgery can be effectively done with this omental plugging procedure. During surgery, margin freshening and biopsy is not performed to perform source control more quickly. This surgical procedure aligned with “quick in-quick out” concept that we adopted for treating patients with gastric perforation. Omental plugging also allows patient to undergo ERAS program for better and faster recovery. The patients were discharged from the hospital without further complications and long-term follow-up showed good results.

**Conclusion:**

Omental plugging has the least risk of complications than other perforation repair techniques and can be done for small and large perforation. Based on our case series, omental plug with double mattress suture is an effective and safe procedure to be performed in elderly patients with gastric perforation with Boey score 3.

## Highlights

• Gastric perforation is life threatening and requires immediate surgical management.
• Omental plugging has the least risk of complications than other perforation repair techniques.
• Omental plugging with double mattress suture is effective and can be done quickly in elderly patients with boey score 3.
• Omental plugging technique allows immediate parenteral feeding and ERAS program, hence better and faster recovery.

## 1 Introduction

Peptic ulcer disease is one of the most common abdominal emergencies in elderly patients, and approximately half of them has develop perforation as complication. The mortality of elderly patients due to gastrointestinal perforation is three times higher than mortality in non-elderly patients. And if the diagnosis and treatment is delayed by 24 h, the mortality rate becomes eight times higher [1]. Physiological functions of body organs are reduced significantly in elderly patients, therefore presence of comorbidities or chronic conditions may increase risk and consequently mortality during and after surgery [2]. Boey score remains to be the simple predictor for mortality and morbidity after surgery despite of varying positive predictive value different studies. Three points in Boey score predicts 38 % mortality rate and 77 % morbidity rate due to gastric perforation [3].

Source control procedure is commonly performed for many complicated tissue infections and especially intra-abdominal infection prone to sepsis [4]. Partial gastrectomy and omental plugging has been the surgery of choice in managing gastric perforation. Other procedures such as serosal patch, partition gastrectomy, or pedicle flap is rarely performed [5]. Omental plugging is one of the most commonly used surgical technique in managing gastric perforation. The procedure involves pulling in the omentum through the perforation and tied using a ryle's tube. Omental plugging is considered safe and reliable for both small and large perforation. It is also associated with less complication compared to omentopexy [6]. This study is reported inline with PROCESS 2020 checklist [7].

## 2 Case presentation

### 2.1 Surgical procedure

**Fig. 1:**
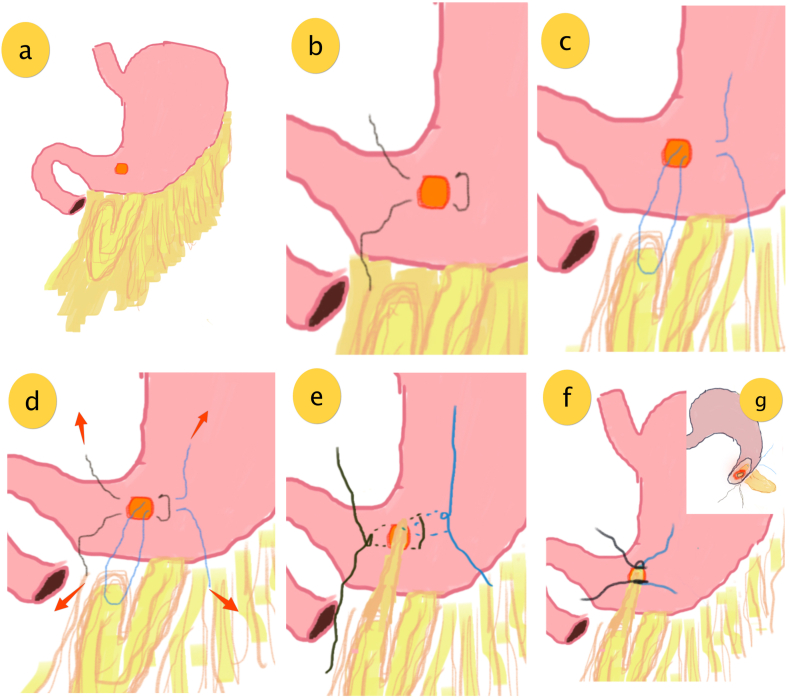
Surgical procedure of omental plugging technique with double mattress suture: **(a)** gastric perforation – orange circle; **(b)** first mattress suture (black line); **(c)** second mattress suture connects gastric wall and a piece of omentum through the perforation (blue line); **(d) the two mattress suture** as well as the omentum section for plugging is established, pulled to shown direction (red arrow); **(e) first mattress suture is** tightened to narrow the perforation site while second mattress suture is tightened to pull the omental plug through the perforation. **(f)** The omentum plug is fixed in place and both mattress suture tail is knotted together with surgeon knot; **(g)** illustration of luminal view of omental plug covering the ulcer and perforation. (For interpretation of the references to colour in this figure legend, the reader is referred to the Web version of this article.)

In these cases, surgery was performed after patient is stabilized from shock. The surgery was performed by a senior gastrointestinal surgeon. All surgeries of presented cases were done in following general steps:

i. We have performed exploratory laparotomy with median incision from xiphoid to umbilicus.
ii. We performed peritoneal lavage with warm NaCl 0,9 % with an amount of approximately 3000–5000 ml.
iii. Gastric perforation (shown as orange circle in Fig. 1) was identified. **(a).**

**Fig. 2:**
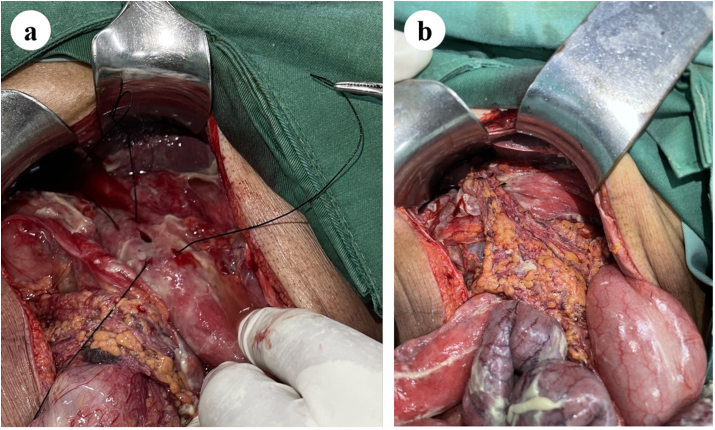
Omental plugging technique with double mattress suture: **(a)** double mattress horizontal suture, each at the cranial and caudal side of the perforation; **(b)** final result of perforation repair with omental plugging technique using double mattress suture.
iv. Mattress suture was made each at the cranial and caudal side of the perforation using horizontal mattress technique with suture thread vicryl 2/0.
v. The first mattress suture (shown as black line in Fig. 1) at the caudal side of perforation was made. **(b)**
vi. The second mattress suture (shown as blue line in Fig. 1) which connects the gastric wall, at the cranial side of the perforation, and a piece of omentum is made. To create a mattress suture end at the omentum, the needle and the thread goes forth and back through the perforation **(c) .**
vii. The second suture is tightened and as a result, the attached omentum was pulled into the perforation hence plugging the perforated site (pull direction shown as red arrow) **(d).**
viii. The first mattress suture was tightened to narrow the perforation and fastening the omentum plug in place, the suture is completed with surgeon knot. The second mattress suture is completed with surgeon knot **(e).**
ix. Both mattress suture tail is knotted together with surgeon knot **(f).**

#### 2.1.1 Case 1

A 75-year-old male is referred from another hospital for emergency surgery with chief complaints of pain throughout the whole abdomen and fever. Patient has been treated for 32 h and stabilized after shock with pre-operative blood pressure of 95/60 mmHg. Patient has diabetes, smoking habit, history of prolonged consumption of analgesic drugs for 2 years, and frequent visits to the doctor for gastric problems. Abdominal x-ray visualized free air in peritoneum and abdominal USG visualized free air in sub-hepatic dan Morrison pouch. Patient is diagnosed with peritonitis caused by GIT perforation with suspicion of gastric perforation.

During emergency laparotomy exploration, gastric antrum perforation was identified and omental pugging with double mattress suture type is performed. Patient underwent Enhanced Recovery after Surgery (ERAS) program with carbohydrate-rich drink in the first two days after surgery, milk in the third day, and *sumsum* porridge (rice flour, coconut milk, and palm sugar) in the fourth and fifth day. Patient was discharged 7 days after surgery in good condition. Long term follow-up showed good results with no further complications.

#### 2.1.2 Case2

An 86-year-old male is referred from another hospital for emergency surgery with chief complaint of pain throughout the whole abdomen and fever. Patient has been treated for 24 h and stabilized after shock with pre-operative blood pressure of 90/74 mmHg. Patient has hypertensive heart disease, history of smoking, and prolonged history of consuming herbal concoctions for his joint pain for the last 1 year. Patient has also been visiting doctor for gastric problems. Physical examination showed signs of distention and free fluid in the abdomen. Abdominal x-ray visualized free air in peritoneal space. Patient is diagnosed with peritonitis caused by GIT perforation with suspicion of gastric perforation.

During emergency laparotomy exploration, gastric antrum perforation was identified and omental pugging with double mattress suture type is performed. Patient underwent Enhanced Recovery after Surgery (ERAS) program with carbohydrate-rich drink in the first two days after surgery, milk in the third day, and *sumsum* porridge (rice flour, coconut milk, and palm sugar) in the fourth and fifth day. Patient was discharged 6 days after surgery in good condition. Long term follow-up showed good results with no further complications.

## 3 Discussion

Perforation is one of the most fatal complications of various disease in gastrointestinal tract. Perforation is most commonly caused by peptic ulcer. Although hemorrhage is still a more common complication than perforation, perforation is an absolute indication for emergency surgery and causes about 40 % of all ulcer-related deaths [8].

The initial treatment of gastric perforation is still a debated topic. According Pieracci and Barie, an effective treatment for intra-abdominal infection is early and proper source control meanwhile according to Marshall, hemodynamic resuscitation becomes the priority treatment which is followed by source control when the patient is stabilized [9,10]. Moreover, based on the guideline by the Surgical Infection Society of America, infection source control should be done regardless because initial resuscitation without successful source control can ultimately lead to septic death [11]. The initiation of source control surgery therefore should not be delayed and the target time for source control and resuscitation must be reached under 6 h since admission. The surgical intervention for source control may significantly raise the outcome of patients with gastrointestinal perforation [12]. In our case, we operated the patients immediately after arrival to the hospital as both patients have been stabilized in other health facilities. The signs of shock also indicate that the perforation might have cause sepsis and therefore requiring immediate source control after resuscitation.

There are various techniques to repair gastric perforation; omental plug, patch, wedge resection, etc. In omental plugging technique, an omental flap is created from the omentum of the patient and about 5–6 cm length of the omental flap is inserted into the gastric perforation. The omentum is then anchored to the perforation with five or six interrupted sutures by using surgical thread silk 2/0. The omentum is then fixed to the healthy duodenum, approximately 3–4 mm away from the perforation site [13]. In our cases, we use two horizontal mattress sutures using surgical thread vicryl 2/0 and half-curved needle with loop. Our omental plugging technique is performed without biopsy and margin freshening to reduce the time taken for perforation repair, thus achieving a quick source control, which aligns with our “quick in-quick out” concept of surgical repair. Gastric perforation repair with only using sutures usually cause tension on the gastric wall which we believe will hinder the healing process of perforation. With omental plug, much less tension is exerted around the perforation. The omental plug also covers the luminal side of the perforation and the ulcers around it, therefore allowing immediate enteral feeding.

For a long period of time, omentopexy is considered the procedure of choice for repairing large size perforations. However, recent studies show that omental plug is considered the better surgical technique for managing large perforations. In cases of large perforation, omental plug carries a lower risk of formation of intestinal fistula when compared to omentopexy. The number of mortality and morbidity following omental plug procedure is also significantly lower when compared to omentopexy. Hence, in cases with small size or large size perforation, omental plug can be considered as the surgery of choice [14]. Omental plugging technique is easy to learn and can avoid major resection of gastrointestinal tract, especially in patients that already have complications [14,15].

Similar to omental plugging technique, omental patch is also one of the studied gastric perforation management technique. However, in the comparative study done in 2016, mortality rate of patients receiving omental plug is 14 % while mortality rate of patients receiving omental patch is 100 % [13]. We believed that omental plugging is a safer and more effective technique in managing gastric perforation compared to other surgical method. This work is still in a case series, therefore, larger sample size is needed to further elaborate the effectiveness of omental plugging with double mattress suture.

## 4 Conclusion

Based on our case series, omental plugging using double horizontal mattress suture is safe and effective on elderly patients with gastric perforation of Boey score 3. This procedure can be quickly performed for emergency source control. The procedure also allows immediate enteral feeding which result in patients able to undergo ERAS program for faster and better recovery.

## Declaration of competing interest

No potential conflict of interest relevant to this article was reported.

## Registration of research studies

The manuscript is a case report, not considered a formal research involving participants.
